# Chemical and microbiological characterization of two traditional Mongolian high‐fat dairy products produced in Xilin Gol, China

**DOI:** 10.1002/fsn3.3283

**Published:** 2023-03-01

**Authors:** Liang Guo, Wei‐Liang Xu, Chun‐Dong Li, Yuan‐Sheng Guo, Mei Ya

**Affiliations:** ^1^ Xilingol Vocational College Xilin Gol Institute of Bioengineering Xilinhot Inner Mongolia China

**Keywords:** characterization, composition, microbiota, traditional dairy products

## Abstract

Mongolian butter and Tude are traditional high‐fat dairy products produced in Xilin Gol, China, which have unique chemical and microbiological characteristics. Mongolian Tude is made from Mongolian butter, dreg, and flour. In this study, the traditional manufacturing process of Mongolian butter and Tude was investigated for the first time. Mongolian butter was characterized by high‐fat content (99.38 ± 0.63%) and high acidity (77.09 ± 52.91°T), whereas Mongolian Tude was considered a high‐fat (21.45 ± 1.23%) and high‐protein (8.28 ± 0.65%) dairy product obtained by butter, dreg, and flour. Mongolian butter and Tude were proven to be safe for human consumption in terms of benzopyrene content. In addition, *Listeria monocytogenes*, *Staphylococcus aureus*, *Salmonella*, coliforms, and aflatoxin M1 were not detected in the samples. Bacteria and molds were not isolated from Mongolian butter; in contrast, the total count of bacteria and molds in Mongolian Tude was within the range of 4.5 × 10^2^ to 9.5 × 10^4^ and 0 to 2.2 × 10^5^, respectively. Moreover, *Lactococcus* (41.55%), *Lactobacillus* (11.05%), *Zygosaccharomyces* (40.20%), and *Pichia* (12.90%) were the predominant bacterial and fungal genera, and *Lactobacillus helveticus* (15.6%), *Lactococcus raffinolactis* (9.6%), *Streptococcus salivarius* (8.5%), *Pantoea vagans* (6.1%), *Bacillus subtilis* (4.2%), *Kocuria rhizophila* (3.5%), *Acinetobacter johnsonii* (3.5%), *Zygosaccharomyces rouxii* (46.2%), *Pichia fermentans* (14.7%), and *Dipodascus geotrichum* (11.7%) were the predominant species in the microbiota of Mongolian Tude. Thus, it can be stated that the microbiota of food products produced by different small families varied significantly. Collectively, the findings presented herein are the first report of chemical and microbiological characterization of products of geographical origin and highlight the need for standardization of manufacturing procedures of Mongolian butter and Tude in the future.

## Graphical abstract

First, the findings presented are the first report of chemical and microbiological characterization of inherited food products (Mongolian butter and Tude) obtained by traditional technologies. Second, nutritional (e.g., fat, protein, moisture, and acidity), safety (e.g., benzopyrene, *Listeria monocytogenes*, *Staphylococcus aureus*, *Salmonella*, coliform, and aflatoxin M1), and microbiological (e.g., TBC, Mold, bacterial, and fungal community) parameters were determined in this study.

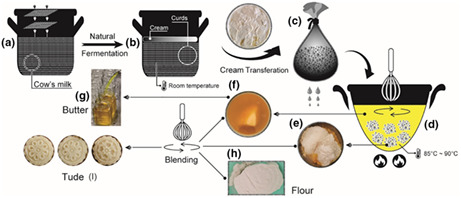

## 1 INTRODUCTION

Mongolian butter produced from cow's milk is a high‐fat dairy product representative of the traditional cuisine of local populations living in Inner Mongolia, Xilingol League, China (Xilingol Traditional Dairy Products Association, 2020). Mongolian butter can be used as a fat component to be mixed with dreg and flour to obtain Mongolian Tude. Both Mongolian butter and Tude play a significant role in Mongolian religious or shamanic celebrations, as Buddhist or Obao offerings. Nevertheless, the traditional processing, as well as the composition and microbiological characteristics (viable microbes and microbiota) of Mongolian butter and Tude have not yet been investigated.

**FIGURE 1:**
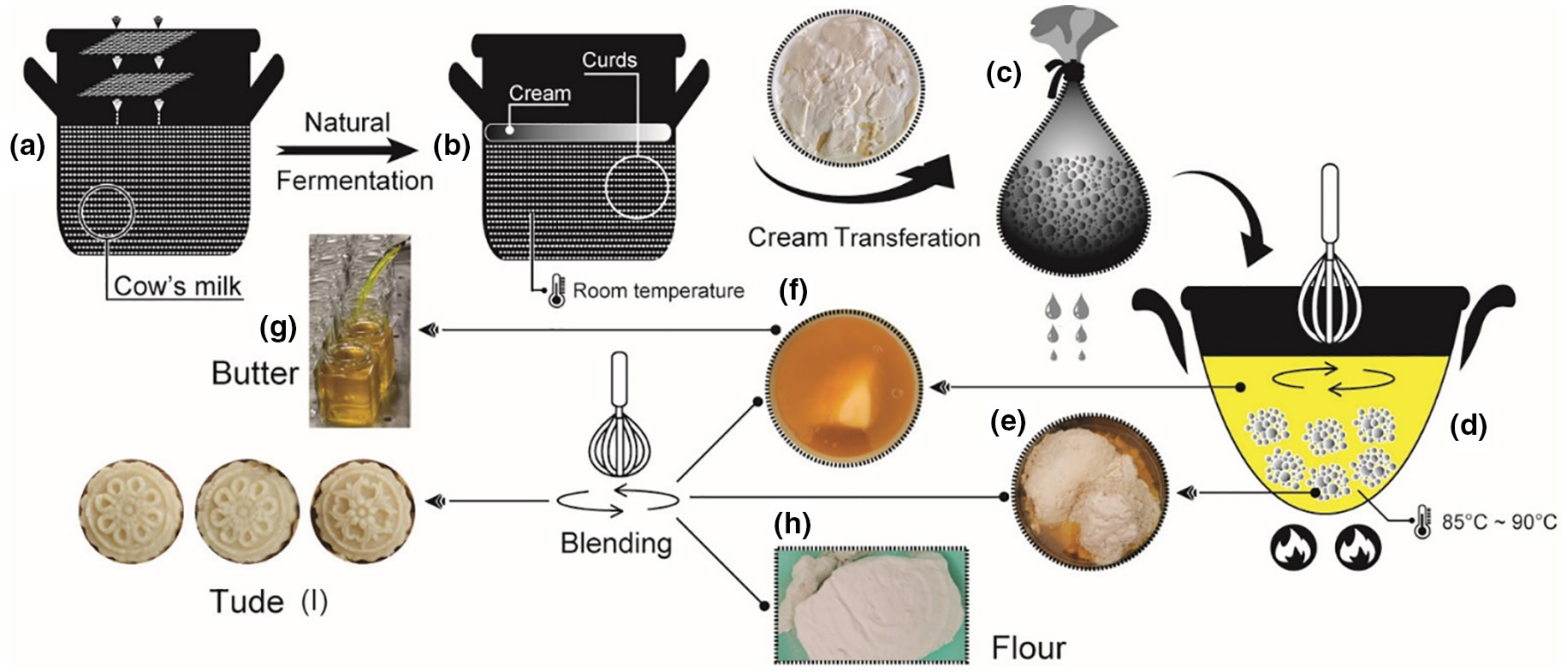
Flow diagram of the traditional manufacturing process of Mongolian butter and Tude.

The traditional processing of Mongolian butter was different from that of commercial butter (Guo & Guo, 2021). To produce the former, filtered fresh milk was spontaneously fermented for 1–2 days (Figure 1a), in which acidity reached 75°T and pH 4.0 at the end of fermentation. Subsequently, the milk surface was covered with a layer of cream (Figure 1b), which was then collected with a spoon and placed inside a fabric bag to allow the draining of excess water (Figure 1c). Then, the cream was transferred to a pot and heated to allow further elimination of residual water (Figure 1d), resulting in a separation into two fractions: the bottom fraction consisted of an oily and acidic substance (dreg; Figure 1e), while the upper fraction was a yellow‐colored transparent butter (Figure 1f). The obtained butter was transferred to a container and stored in a cool place (Figure 1g). For preparing Mongolian Tude, wheat flour was toasted in an oven (Figure 1h) and then mixed with butter and dreg (at a ratio of 1:1:1, *w/w*) to obtain a slightly elastic dough, which was subsequently placed in molds (Figure 1i). After processing, Mongolian butter and Tude can undergo long‐term storage by refrigeration, during which special organoleptic qualities were developed.

Considering the steps used in the processing of Mongolian butter and Tude, it can be concluded that spontaneous/natural fermentation and cooling storage are the main factors that are responsible for the development of special properties of these products, such as fermented smell, sourness, the taste of milk with wheat aroma. However, since this artisanal process is based on natural fermentation, it may comport risks for food safety due to the contamination with pathogenic bacteria and molds. In addition, mycotoxin production and/or benzopyrene formation can occur, respectively, as a result of mold growth and excessive temperature.

Thus, the present study aimed to comprehensively evaluate the chemical composition and microbiological safety of traditional Mongolian butter and Tude based on chemical, microbiological, and microbial community analyses.

## 2 MATERIALS AND METHODS

### 2.1 Mongolian butter and Tude collection

Mongolian butter and Tude samples were collected in Xilin Gol, China. A total number of 42 samples of butter were obtained from Mongolian family‐sized production plants resided in Xilinhot, West Ujimqin Banner, East Ujimqin Banner, East Sunit Banner, Abag Banner, Huang Banner, and Lan Banner, respectively. A total number of 10 samples of Tude were obtained from Mongolian production plants resided in Huang Banner and Lan Banner, respectively. These handmade dairy products were immediately stored at 4°C and analyzed to investigate the chemical composition, viable microbes, and microbial community.

### 2.2 Determination of fat, solids‐not‐fat, protein, moisture, benzopyrene, aflatoxin M1 contents, and acidity

The fat (China National Food Safety Standard, 2016a), solids‐not‐fat (China National Food Safety Standard, 2010), protein (China National Food Safety Standard, 2016b), moisture (China National Food Safety Standard, 2016c), benzopyrene (China National Food Safety Standard, 2016d), and aflatoxin M1 content (China National Food Safety Standard, 2016e) were determined according to the Chinese national food safety standards. The Soxhlet extraction method was used to measure fat content. Solids‐not‐fat was determined by subtracting percent fat from percent total solids. The Kjeldahl method was used to measure protein content. Benzopyrene and aflatoxin M1 were determined by the HPLC method. Acidity was determined by the titration method using sodium hydroxide (0.1 mol/L) with a phenolphthalein indicator (Sinopharm; China National Food Safety Standard, 2016f). The data obtained from the experiment were analyzed by ANOVA in SPSS20.0.

### 2.3 Quantification of coliform, TBC, Mold, *Listeria monocytogenes*, *Staphylococcus aureus*, and S*almonella*

The enumeration of coliforms was determined using Violet Red Bile Agar (VRBA) and Brilliant Green Lactose Bile (BGLB) for 48 h at 36°C (China National Food Safety Standard, 2016g). TBC (China National Food Safety Standard, 2016h) was calculated using Plant Count Agar at 36°C for 2 days. The mold (China National Food Safety Standard, 2016i) was calculated using Rose Bengal Agar with chloramphenicol at 28°C for 5 days. *L. monocytogenes* (China National Food Safety Standard, 2016j), *S. aureus* (China National Food Safety Standard, 2016k), and *Salmonella* (China National Food Safety Standard, 2016l) were detected according to the protocols in these relevant China National Food Safety Standards. The data obtained from the experiment were analyzed by ANOVA in SPSS20.0.

### 2.4 16 S rRNA and ITS gene sequencing, bioinformatics, and statistical analysis

The total DNA was purified using the E.Z.N.A DNA kit (Omega Bio‐tek). The V3‐V4 region of *16 S rRNA* gene was amplified by using 341F 5′‐CCTACGGGNGGCWGCAG‐3′ and 806R 5′‐GGACTACHVGGGTATCTAAT‐3′ primers. The *ITS* gene was amplified by using ITS3‐KYO2F 5′‐GATGAAGAACGYAGYRAA‐3′ and ITS4R 5′‐TCCTCCGCTTATTGATATGC‐3′. The PCR reaction included 1 μL of KOD polymerase, 5 μL of 2.5 mM dNTPs, 5 μL of 10× KOD buffer, 1.5 μL of each primer (5 μM), and 100 ng of DNA. The program of PCR reaction was as follows: initial denaturation at 95°C for 2 min; followed by 27 cycles of denaturation at 98°C for 10 s, annealing at 62°C for 30 s, and extension at 68°C for 30 s; and final extension at 68°C for 10 min. The amplicons were quantified and subjected to paired‐end sequencing (2 × 250) using the Illumina MiSeq platform (Illumina). The final reads were obtained by trimming the chimeric tags and were clustered into the operational taxonomic units (OTUs) by using the UPARSE pipeline (Edgar, 2013). The OTUs were classified into organisms with the Naive Bayesian Model by using RDP classifier (Wang et al., 2007) based on the SILVA database for *16 S rRNA* gene sequencing (Pruesse et al., 2007) and UNITE database for *ITS* gene sequencing (Koljalg et al., 2005).

## 3 RESULTS

### 3.1 Chemical composition of Mongolian butter and Tude

As shown in Table 1, the contents of fat and solids‐not‐fat in Mongolian butter were 99.38 ± 0.63% and 0.46 ± 0.64%, respectively; the moisture content was 0.16 ± 0.09%. Overall, no significant difference was found in the evaluated parameters (fat, solids‐not‐fat, and moisture) between different producing regions (*p* > .05). Nevertheless, the acidity of Mongolian butter produced in different producing regions varied significantly (*p* < .05). Although Mongolian butter is derived from naturally fermented cream, its composition is roughly identical to commercial butter. However, the acidity of Mongolian butter is 77.09 ± 52.91°T, which is significantly higher than that of commercial butter. High acidity confers unique qualities to butter, such as distinct flavor, and also acts as a natural preservative.

**TABLE 1:** Chemical characterization of Mongolian butter from Xilin Gol, China.

Sample	Location	Fat (g/100 g)	Solids‐not‐fat (g/100 g)	Moisture (g/100 g)	Acidity (°T)
Butter 1–6	Xilinhot	99.68 ± 0.17	0.21 ± 0.12	0.11 ± 0.07	37.40 ± 13.96
Butter 7–12	West Ujimqin Banner	99.33 ± 0.72	0.50 ± 0.67	0.17 ± 0.08	139.38 ± 81.86
Butter 13–18	East Ujimqin Banner	99.15 ± 0.83	0.65 ± 0.81	0.20 ± 0.04	103.02 ± 3.83
Butter 19–24	East Sunit Banner	98.75 ± 0.85	1.18 ± 0.85	0.07 ± 0.03	35.28 ± 20.91
Butter 25–30	Abag Banner	99.57 ± 0.22	0.28 ± 0.29	0.15 ± 0.09	66.92 ± 35.22
Butter 31–36	Huang Banner	99.47 ± 0.32	0.32 ± 0.29	0.22 ± 0.13	89.43 ± 58.15
Butter 37–42	Lan Banner	99.70 ± 0.15	0.10 ± 0.13	0.20 ± 0.07	68.22 ± 13.30

Mongolian Tude is produced from Mongolian butter, dreg, and flour, thus containing milk‐like and wheat‐like odors. As shown in Table 2, the contents of fat and protein in Mongolian Tude were 21.45 ± 1.23% and 8.28 ± 0.65%, respectively, and its moisture content was 17.21 ± 2.30%. The content of protein in Mongolian Tude from Huang Banner was significantly higher than that from Lan Banner (*p* < .05), and no significant difference was found in the contents of fat and moisture between Huang Banner and Lan Banner (*p* > .05). Flour from farming areas is an extremely valuable product to nomadic populations, which is used in the mixture of Mongolian butter and dreg to produce Mongolian Tude. Mongolian Tude has a strong milk taste with wheat flavor which results from the fusion of nomadic and agriculture‐based populations. Specifically, Mongolian nomadic groups use Mongolian Tude as an offering in Buddhist celebrations and Obao folk religious rituals.

**TABLE 2:** Chemical characterization of Mongolian Tude in Xilin Gol, China.

Sample	Location	Fat (g/100 g)	Protein (g/100 g)	Moisture (g/100 g)
Tude 1	Huang Banner	23.3	9.29	22.8
Tude 2	Huang Banner	22.7	8.26	17.6
Tude 3	Huang Banner	21.1	9.49	19
Tude 4	Huang Banner	20.7	7.71	16
Tude 5	Huang Banner	20.4	7.76	15.7
Tude 6	Lan Banner	20.3	8.5	15.4
Tude 7	Lan Banner	20.9	7.88	15.6
Tude 8	Lan Banner	21.1	7.69	15.8
Tude 9	Lan Banner	20.5	7.93	16.3
Tude 10	Lan Banner	23.5	8.33	17.9

### 3.2 Evaluation of chemical and microbiological safety of Mongolian butter and Tude

The chemical and microbiological safety of Mongolian butter and Tude has been proven by their long history of production and consumption. However, natural fermentation, high temperature, family‐sized production, and the absence of preservatives increase the risk associated with the consumption of Mongolian butter and Tude, mainly involving pathogenic bacteria and molds, and benzopyrene formation. As shown in Table 3, benzopyrene content was 0 in Mongolian butter. In contrast, benzopyrene was found in two out of 10 samples of Mongolian Tude (1.7 and 1.2 μg/kg), at levels below the criteria established in European food safety standards (2 μg/kg). The above results indicated that the high temperature employed during the production of Mongolian butter did not lead to benzopyrene formation. However, dreg used in the production of Mongolian butter may contain traces of benzopyrene, especially in the portions on the bottom of the pot.

**TABLE 3:** Evaluation of food safety of Mongolian butter and Tude in Xilin Gol, China.

Sample	Location	Benzopyrene (μg/kg)	Coliform (CFU/g)	TBC (CFU/g)	Mold (CFU/g)	Aflatoxin M1 (μg/kg)
Butter 1–6	Xilinhot	0	0	0	0	0
Butter 7–12	West Ujimqin Banner	0	0	0	0	0
Butter 13–18	East Ujimqin Banner	0	0	0	0	0
Butter 19–24	East Sunit Banner	0	0	0	0	0
Butter 25–30	Abag Banner	0	0	0	0	0
Butter 31–36	Huang Banner	0	0	0	0	0
Butter 37–42	Lan Banner	0	0	0	0	0
Tude 1	Huang Banner	0	10	2.8 × 10^4^	0	0
Tude 2	Huang Banner	0	0	3.0 × 10^3^	50	0
Tude 3	Huang Banner	0	0	9.5 × 10^4^	2.7 × 10^2^	0
Tude 4	Huang Banner	0	0	8.6 × 10^2^	1.1 × 10^3^	0
Tude 5	Huang Banner	0	0	1.6 × 10^3^	9.9 × 10^2^	0
Tude 6	Lan Banner	0	0	1.2 × 10^3^	1.9 × 10^2^	0
Tude 7	Lan Banner	0	0	1.7 × 10^3^	2.0 × 10^3^	0
Tude 8	Lan Banner	1.7	0	4.5 × 10^2^	3.7 × 10^2^	0
Tude 9	Lan Banner	1.2	0	9.4 × 10^4^	2.2 × 10^5^	0
Tude 10	Lan Banner	0	5	9.7 × 10^2^	2.7 × 10^2^	0

Considering microbiological analysis, *L. monocytogenes*, *S. aureus*, and *Salmonella* could not be detected in none of the Mongolian butter and Tude samples. As shown in Table 3, coliform counts in Mongolian butter were 0, whereas low coliform counts were detected in two out of 10 Tude samples. Family‐sized environment for Tude production might pose a certain degree of food safety risk. Moreover, due to the high temperature employed in the manufacture of Mongolian butter, bacteria and molds were not detected. In contrast, as shown in Table 3, total counts of bacteria and molds were determined in Tude. The count of mold in Mongolian Tude from Huang Banner was significantly lower than that from Lan Banner (*p* < .05), and no significant difference was found in the total count of bacteria between Huang Banner and Lan Banner (*p* > .05). Nonsterile conditions found in family‐sized production environment and equipment may serve as contamination sources of bacteria and molds in Tude, and its chemical composition of high‐fat and high‐protein contents with moderate moisture content is suitable for the growth of bacteria and molds. Although molds were detected in Tude, aflatoxin M1 could not be found in none of the samples. Collectively, Mongolian butter and Tude were shown to be safe for consumption based on the findings of the current study.

### 3.3 Composition of the bacterial community of Mongolian Tude

**FIGURE 2:**
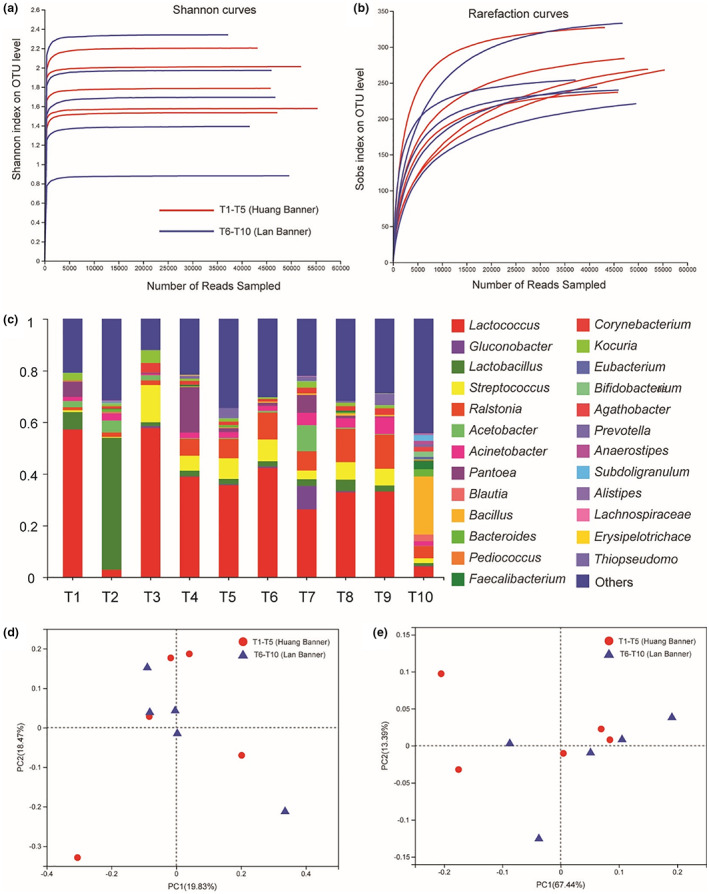
Shannon diversity curves (a) and Rarefaction curves (b) for 16 S rRNA sequencing analysis. Relative abundance of bacterial sequences at genus level in the 10 individual Tude samples obtained from Mongolian family‐sized production plants resided in Huang Banner and Lan Banner (c). Unweighted (d) and weighted (e) UniFrac principal coordinate analyses of the bacterial diversities in the 10 individual Tude samples. OTU, operational taxonomic units.

After the removal of low‐quality reads, 472,147 reads (Average ± SD: 47,215 ± 5703) assigned to bacteria were identified in Tude collected from 10 family‐sized production sites. OTUs, as well as Shannon, Simpson, Chao1, and Good's coverage indices, were used to evaluate the richness and diversity of bacterial communities. Shannon curves (Figure 2a) but not rarefaction curves (Figure 2b) achieved data saturation in *16 S rRNA* gene sequencing (Table 4). Thus, the above results indicated that sequencing depth was sufficient to adequately represent bacterial communities in samples. No significant differences were found in the diversity of bacterial communities of Mongolian Tude samples from two producing regions (*p* > .05).

**TABLE 4:** Diversity indices of amplicon sequencing of Mongolian Tude in Xilin Gol, China.

Target	Sample	No. of reads	No. of OTU	Shannon index	Simpson index	Chao1 index	Good's coverage
Bacterial analysis	Tude 1	58,054	268	1.58	0.47	331	0.9988
	Tude 2	44,057	327	2.20	0.33	334	0.9996
	Tude 3	52,693	269	2.01	0.26	292	0.9991
	Tude 4	46,343	237	1.79	0.43	247	0.9996
	Tude 5	47,874	284	1.54	0.55	312	0.9993
	Tude 6	46,720	240	1.97	0.38	245	0.9997
	Tude 7	41,681	244	1.39	0.58	263	0.9992
	Tude 8	49,982	221	0.88	0.74	254	0.9992
	Tude 9	47,303	333	1.69	0.51	341	0.9995
	Tude 10	37,440	254	2.34	0.36	265	0.9996
Fungal analysis	Tude 1	65,446	140	2.37	0.14	152	0.9997
	Tude 2	56,397	52	2.18	0.16	60	0.9999
	Tude 3	64,589	118	2.59	0.15	126	0.9998
	Tude 4	71,528	91	2.62	0.13	94	0.9999
	Tude 5	55,899	81	2.39	0.14	84	0.9999
	Tude 6	69,296	90	2.56	0.12	92	0.9999
	Tude 7	39,351	97	2.61	0.12	102	0.9998
	Tude 8	70,656	73	1.11	0.57	79	0.9998
	Tude 9	42,373	53	0.72	0.76	64	0.9997
	Tude 10	50,106	48	2.80	0.10	49	0.9999

Based on the composition and abundance of bacterial communities at the phylum level, Firmicutes, Proteobacteria, Actinobacteria, and Bacteroidetes represented 52.42%, 34.49%, 7.38%, and 3.52% of bacteria in Tude samples, respectively. As shown in Figure 2c, ten bacterial genera (>1%) were identified in Huang Banner Tude, including *Lactococcus* (44.83%), *Lactobacillus* (16.37%), *Streptococcus* (7.72%), *Pantoea* (4.95%), *Ralstonia* (3.79%), *Kocuria* (2.74%), *Acetobacter* (2.42%), *Corynebacterium* (2.07%), *Acinetobacter* (1.99%), and *Chryseobacterium* (1.19%), whereas 18 bacterial genera (>1%) were identified in Lan Banner Tude, including *Lactococcus* (36.49%), *Ralstonia* (12.31%), *Bacillus* (7.40%), *Streptococcus* (7.19%), *Acinetobacter* (4.56%), *Lactobacillus* (2.82%), *Acetobacter* (2.42%), *Gluconobacter* (2.19%), *Pantoea* (2.09%), *Staphylococcus* (1.97%), *Pseudomonas* (1.95%), *Corynebacterium* (1.80%), *Enhydrobacter* (1.42%), *Kocuria* (1.27%), *Delftia* (1.25%), *Leuconostoc* (1.15%), *Chryseobacterium* (1.09%), and *Rhodococcus* (1.04%). At the species level, 13 bacterial species (>1%) were identified in Huang Banner Tude, including *Lactobacillus helveticus* (24.39%), *Streptococcus salivarius* (9.43%), *Pantoea vagans* (8.10%), *Lactococcus raffinolacti*s (8.00%), *Kocuria rhizophila* (4.48%), *Acinetobacter johnsonii* (2.27%), *Lactococcus garvieae* (2.04%), *Streptococcus equinus* (1.89%), *Triticum aestivum* (1.32%), *Corynebacterium flavescens* (1.25%), *Leuconostoc mesenteroides* (1.23%), *Moraxella osloensis* (1.19%), and *Staphylococcus aureus* (1.12%), whereas 20 bacterial species (>1%) were identified in Lan Banner Tude, including *Lactococcus raffinolactis* (12.09%), *Bacillus subtilis* (10.61%), *Streptococcus salivarius* (7.37%), *Acinetobacter johnsonii* (5.32%), *Gluconobacter oxydans* (3.45%), *Pantoea vagans* (3.33%), *Staphylococcus aureus* (2.96%), *Lactobacillus helveticus* (2.74%), *Moraxella osloensis* (2.26%), *Lactococcus garvieae* (2.08%), *Kocuria rhizophila* (2.03%), *Delftia tsuruhatensis* (1.98%), *Leuconostoc mesenteroides* (1.73%), *Escherichia coli* (1.71%), *Faecalibacterium prausnitzii* (1.66%), *Rhodococcus erythropolis* (1.63%), *Anoxybacillus flavithermus* (1.53%), *Streptococcus equinus* (1.30%), *Pseudomonas psychrophila* (1.21%), and *Acinetobacter guillouiae* (1.14%). Most genera and species found in Tude samples have been previously identified in naturally fermented dairy products produced in Xilin Gol, China (Guo et al., 2019, 2021; Yamei et al., 2019). Nevertheless, certain bacterial genera (e.g., *Pantoea*, *Ralstonia*, *Kocuria*, and *Bacillus*) were described for the first time in Tude, but were not found in traditional koumiss (Guo et al., 2019), traditionally fermented vrum (Yamei et al., 2019), Khoormog, Chigee, and Airag produced in Xilin Gol (Guo et al., 2021). In addition, a higher number of bacterial genera and species in Tude was found compared to other naturally fermented dairy products. Considering that Tude contains flour, it can be easily contaminated with a wider variety of microorganisms.

Furthermore, multivariate analysis was used to compare the composition and abundance of bacteria communities of Mongolian Tude from two producing regions. Figure 2d depicts unweighted PCoA and Figure 2e depicts weighted PCoA at genus‐level OTUs of *16 s rRNA* gene sequencing. No significant differences were found in the bacterial communities of Tude samples from two producing regions (ANOSIM: *R* = −0.008, *p* = .524 for unweighted PCoA; *R* = −0.08, *p* = .721 for weighted PCoA), thus indicating that the producing region did not affect composition and abundance of bacteria in Tude.

### 3.4 Composition of fungal community of Mongolian Tude

**FIGURE 3:**
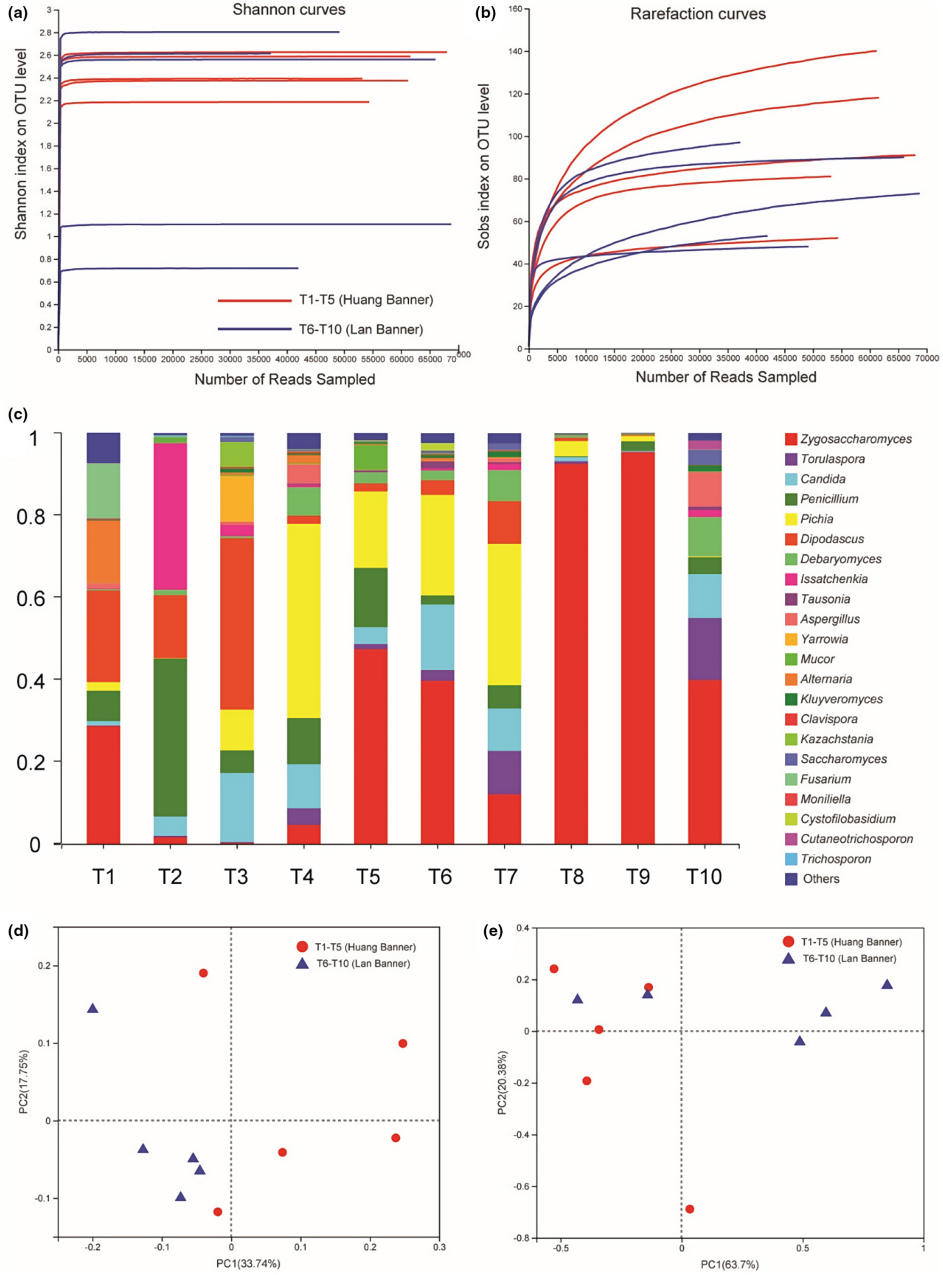
Shannon diversity curves (a) and Rarefaction curves (b) for ITS sequencing analysis. Relative abundance of fungal sequences at genus level in the 10 individual Tude samples obtained from Mongolian family‐sized production plants resided in Huang Banner and Lan Banner (c). Unweighted (d) and weighted (e) UniFrac principal coordinate analyses of the fungal diversities in the 10 individual Tude samples. OTU, operational taxonomic units.

After the removal of low‐quality reads, a total of 585,641 reads (Average ± SD: 58,564 ± 11,680) assigned to fungi were generated from Tude samples collected from ten family‐sized production sites. OTUs, as well as Shannon, Simpson, Chao1, and Good's coverage indices, were used to evaluate the richness and diversity of fungal communities in samples. Shannon curves (Figure 3a) but not rarefaction curves (Figure 3b) enabled data saturation in *ITS* gene sequencing (Table 4). Collectively, the results demonstrated that sequencing depth was sufficient to adequately represent fungal communities in samples. No significant differences were found in the diversity of fungal communities in Mongolian Tude from two producing regions (*p* > .05).

Considering the composition and abundance of fungi at the phylum level, Ascomycota and Basidiomycota represented 97.68% and 1.63% of the fungal community in Tude samples, respectively. As shown in Figure 3c, 14 fungal genera (>1%) were identified in Huang Banner Tude, including *Dipodascus* (18.37%)*, Pichia* (17.49%)*, Penicillium* (15.52%)*, Zygosaccharomyces* (12.35%)*, Candida* (9.22%)*, Issatchenkia* (8.45%)*, Yarrowia* (3.29%)*, Debaryomyces* (2.54%)*, Alternaria* (2.13%)*, Kazachstania* (1.74%)*, Fusarium* (1.46%)*, Aspergillus* (1.44%)*, Mucor* (1.37%)*, and Torulaspora* (1.34%), whereas nine fungal genera (>1%) were identified in Lan Banner Tude, including *Zygosaccharomyces* (65.13%), *Pichia* (8.79%), *Candida* (6.58%), *Torulaspora* (4.88%), *Debaryomyces* (3.35%), *Penicillium* (2.31%), *Aspergillus* (1.99%), *Dipodascus* (1.79%), and *Saccharomyces* (1.06%). The composition and abundance of fungi at the species level revealed that 14 fungal species (>1%) were identified in Huang Banner Tude, including *Dipodascus geotrichum* (23.01%), *Pichia fermentans* (19.74%), *Zygosaccharomyces rouxii* (15.24%), *Issatchenkia orientalis* (10.58%), *Candida pararugosa* (4.93%), *Yarrowia lipolytica* (4.12%), *Candida zeylanoides* (2.86%), *Alternaria tenuissima* (2.59%), *Pichia cactophila* (2.10%), *Kazachstania servazzii* (2.02%), *Fusarium asiaticum* (1.76%), *Mucor racemosus* (1.71%), *Candida sorbosivorans* (1.46%), and *Aspergillus tubingensis* (1.42%), whereas six fungal species (>1%) were identified in Lan Banner Tude, including *Zygosaccharomyces rouxii* (72.60%), *Pichia fermentans* (10.35%), *Candida zeylanoides* (4.25%), *Dipodascus geotrichum* (2.14%), *Aspergillus tubingensis* (1.11%), *and Candida mesenterica* (1.11%). Compared with other naturally fermented dairy products from Xilin Gol, China (Guo et al., 2019, 2021; Yamei et al., 2019), the diversity of fungal genera and species in Mongolian Tude was notably higher, and the relative abundance of certain fungi was increased. Considering the family‐sized production conditions and nutrient‐rich matrix, a wide variety of molds can grow in Tude. Fungal contamination in traditional dairy products obtained by natural fermentation is common, which may also confer distinct flavors to these dairy products.

In addition, multivariate analysis was used to compare the composition and abundance of fungal communities in Mongolian Tude samples from two producing regions. Figure 3d depicts unweighted PCoA, whereas weighted PCoA is shown in Figure 3e based on genus‐level OTUs obtained with *ITS* gene sequencing. No significant differences were found in the composition of fungal communities between Tude samples from two producing regions (ANOSIM: *R* = 0.328, *p* = .017 for unweighted PCoA; *R* = 0.228, *p* = .11 for weighted PCoA). Thus, it can be stated that the microbiota of Tude did not differ based on producing region.

## 4 CONCLUSIONS

Mongolian butter and Tude are traditional high‐fat dairy products, and their traditional processing steps, chemical composition, and microbiological characteristics have not been reported so far. In this study, nutritional composition (e.g., fat, protein, moisture, and acidity), safety (e.g., benzopyrene, *L. monocytogenes*, *S. aureus*, *Salmonella*, coliform, and aflatoxin M1), and microbiological (e.g., TBC, mold, bacterial and fungal community) parameters were determined. Overall, Mongolian butter could be considered a traditional acidic butter with fermented odor, whereas Tude could be characterized as a flour‐containing high‐fat and high‐protein suspension. Although natural fermentation is employed in the manufacture of Mongolian butter and Tude in an artisanal and family‐sized production environment which could potentially aggravate food safety risks, Mongolian butter and Tude could be considered safe food products to a certain extent.

## CONFLICT OF INTEREST STATEMENT

All authors declare that they have no conflict of interest.

## ETHICS STATEMENT

This study does not involve any human or animal testing.

## Data Availability

The data that support the findings of this study are available on request from the corresponding author.
